# Many Voices in a Choir: Tumor-Induced Neurogenesis and Neuronal Driven Alternative Splicing Sound Like Suspects in Tumor Growth and Dissemination

**DOI:** 10.3390/cancers13092138

**Published:** 2021-04-29

**Authors:** Zodwa Dlamini, Kgomotso Mathabe, Llewellyn Padayachy, Rahaba Marima, George Evangelou, Konstantinos N. Syrigos, Arianna Bianchi, Georgios Lolas, Rodney Hull

**Affiliations:** 1 SAMRC Precision Prevention and Novel Drug Targets for HIV-Associated Cancers (PPNDTHAC) Unit, Pan African Cancer Research Institute (PACRI), University of Pretoria, Hatfield 0028, South Africa; kgomotso.mathabe@up.ac.za (K.M.); lc.padayachy@up.ac.za (L.P.); rahaba.marima@up.ac.za (R.M.); glolas@med.uoa.gr (G.L.); rodney.hull@up.ac.za (R.H.); 2 Department of Urology, University of Pretoria, Pretoria 0084, South Africa; 3 Department of Neurosurgery, University of Pretoria, Pretoria 0084, South Africa; 4 3rd Department of Medicine, National & Kapodistrian University of Athens, 11527 Athens, Greece; grgevangelou@med.uoa.gr (G.E.); ksyrigos@med.uoa.gr (K.N.S.); 5 Liceo Scientifico Statale “G. Galilei”, 53100 Siena, Italy; arianna.bianchi@galilei.edu.it

**Keywords:** neoneurogenesis, nerves, alternative splicing, cancer growth and development, therapeutic targets

## Abstract

**Simple Summary:**

Significant progress has recently been made in understanding the role of the neuronal system in cancer biology, in many solid tumors such as prostate, breast, pancreatic, gastric and brain cancers. Solid tumors and the nervous system appear to influence each other’s development both directly and indirectly. A recurring element in such interactions is constituted by nerve-related substances such as neurotransmitters and neurotrophins, to which the first part of the current review is devoted. The second part of the review focuses on the potential role played by alternative splicing in cancer progression associated with neural signaling. Alternative splicing is the process where pre-mRNA is cut and re-ligated in different ways to give rise to multiple protein isoforms whose expression profile is often cancer specific. Alternative splicing is known to take place in the mRNA of genes that code for proteins involved in neuronal development and the creation of new nerve fibers. The change in alternative splicing patterns that occur as tumors develop and progress may make these splice variants potential targets for the development of drug treatments. They may also serve as diagnostic or prognostic biomarkers.

**Abstract:**

During development, as tissues expand and grow, they require circulatory, lymphatic, and nervous system expansion for proper function and support. Similarly, as tumors arise and develop, they also require the expansion of these systems to support them. While the contribution of blood and lymphatic systems to the development and progression of cancer is well known and is targeted with anticancer drugs, the contribution of the nervous system is less well studied and understood. Recent studies have shown that the interaction between neurons and a tumor are bilateral and promote metastasis on one hand, and the formation of new nerve structures (neoneurogenesis) on the other. Substances such as neurotransmitters and neurotrophins being the main actors in such interplay, it seems reasonable to expect that alternative splicing and the different populations of protein isoforms can affect tumor-derived neurogenesis. Here, we report the different, documented ways in which neurons contribute to the development and progression of cancer and investigate what is currently known regarding cancer-neuronal interaction in several specific cancer types. Furthermore, we discuss the incidence of alternative splicing that have been identified as playing a role in tumor-induced neoneurogenesis, cancer development and progression. Several examples of changes in alternative splicing that give rise to different isoforms in nerve tissue that support cancer progression, growth and development have also been investigated. Finally, we discuss the potential of our knowledge in alternative splicing to improve tumor diagnosis and treatment.

## 1. Overview of Tumor-Nerves Interactions’ Main Components

### 1.1. The Structure and Signaling of the Autonomic Nervous System

The Autonomic Nervous System (ANS, also called the “visceral nervous system” or the “involuntary nervous system”) is made of the nerves and the ganglia (i.e., nerve clusters) that control body visceral functions below the level of consciousness (such as heart and respiratory rates, digestion and pupillary dilation) [1]. This is made of three sub-systems:

- The sympathetic nervous system (SNS), or “fight or flight” system, which is generically responsible for quick response processes (e.g., vasoconstriction to divert blood flow, dilation of the respiratory ducts, heart rate increase).
- The parasympathetic nervous system (PNS), or “rest and digest” system, which governs slower responses (e.g., vasodilation for gastro-intestinal functions, stimulation of saliva secretion); and
- The enteric nervous system (ENS), or “feed and breed” system, which mainly controls the function of the gastrointestinal system.

**Figure 1:**
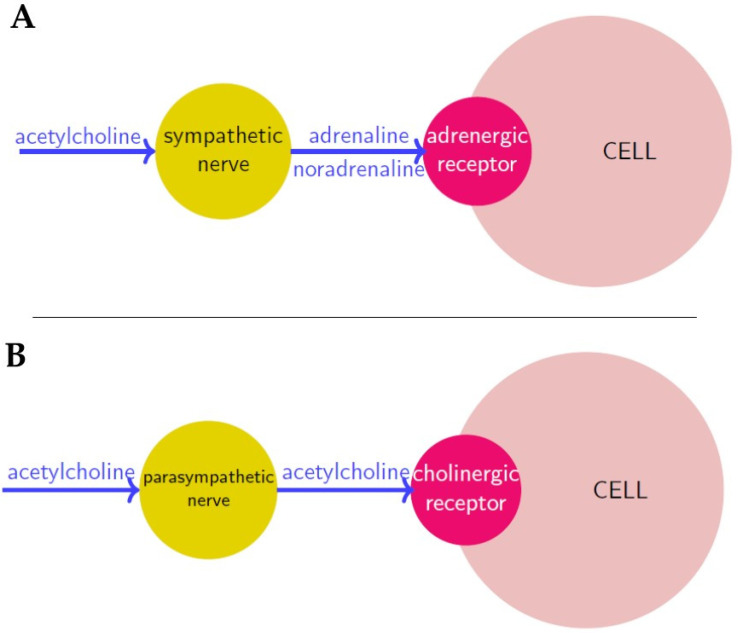
Neurosignaling in sympathetic (**A**) and parasympathetic (**B**) innervation respectively. Neurotransmitters are in blue, nerves are in yellow and receptors in red.

The SNS is constituted by ganglia which are parallel to the spinal cord and spread from the first thoracic vertebra to the second/third lumbar vertebra, while the PNS mainly consists of the vagus nerve (the X cranial nerve) and some spinal nerves (the pelvic splanchnic nerve). Ganglia of both systems respond to a neurotransmitter called *acetylcholine*, but only parasympathetic ones produce it; sympathetic ganglia instead release *adrenaline* (or *epinephrine*) and *noradrenaline* (or *norepinephrine*). The actions that these substances perform depend on the receptor type they bind to. Adrenaline and noradrenaline bind to α- and β-receptors (*adrenergic receptors*), while acetylcholine uses muscarinic and nicotinic receptors (*cholinergic receptors*)—see Figure 1.

Nerve cells or neurons propagate information through electro-chemical impulses. These impulses travel along the axons, as such the axons are referred to as called *nerve fibres*. Two neurons exchange information across a synapse through chemicals (the so-called *neurotransmitters*), which are a “translation” of the electrical signals travelling inside the neurons. Finally, a neurotransmitter released by a nerve binds to a receptor on another cell, and according to the receptor type, it induces a certain action.

Finally, nerves can be classified based on their cellular origin [2]. Those that originate in the spinal column or brain stem are known as the central nervous system or the *pre-ganglion* neuron series. Those that originate in the periphery of the ganglion are known as the *post-ganglion* neural series.

### 1.2. Tumor-Nerves Reciprocal Effects

**Figure 2:**
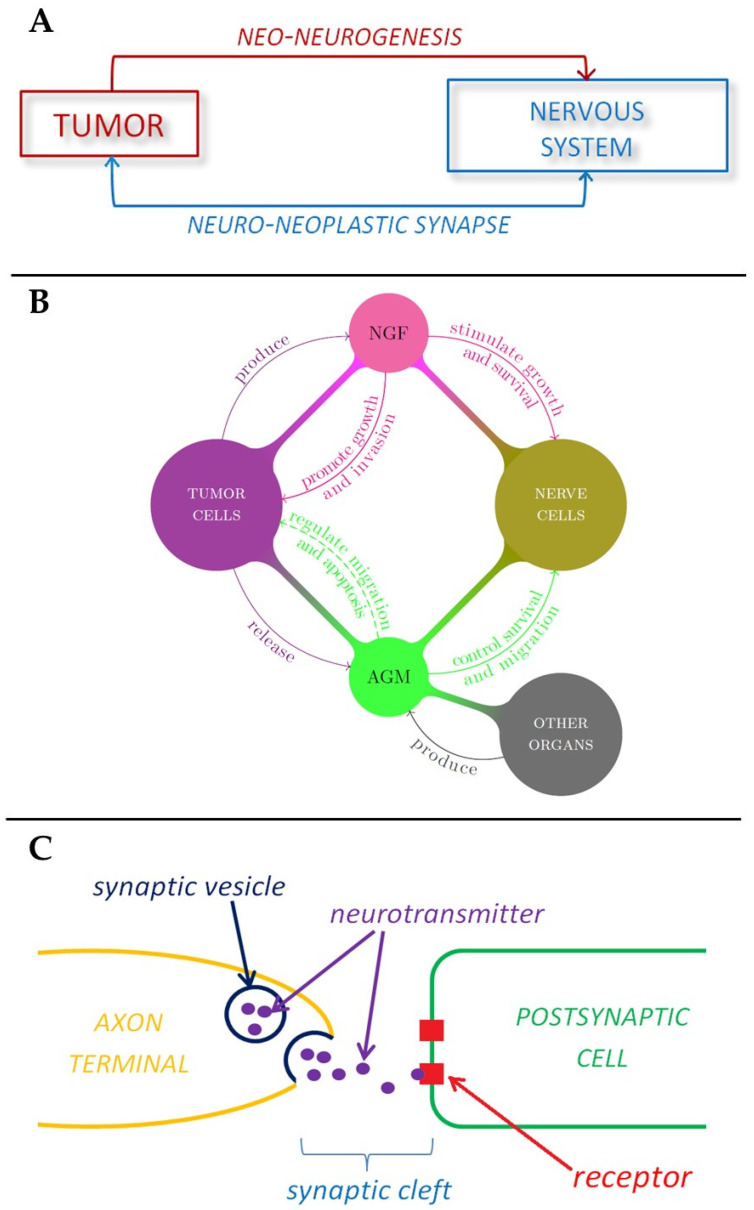
Schematic representations of the role played by nervous system in tumor development. (**A**) Tumor-nerve bi-directional interaction. (**B**) The role of nerve growth factors and axon guidance molecules in tumor-nerve relationship. (**C**) The mechanism of neurotransmitter signaling in a synapse.

A relationship between tumors and the nervous system has been suspected for a long time (since Galen in the 2nd century AD) [3], because of several observations such as the effects of stress on cancer progression, the high innervation of tumor tissues, or the influence that neurotransmitters have on tumorigenesis [4,5]. New evidence strongly suggests the neuronal system is a key player in cancer initiation, progression and dissemination [5,6]. It is believed that in the same way that the nervous system can influences growth, development, and maintenance in normal tissue [7,8,9], it can contribute to the development and spread of cancer [5,10,11]. As such the formation of new neural tissue has been identified as a hallmark of cancer and can be correlated with cancer severity [12,13]. The connection between the ANS and tumors is bilateral, in the sense that on one hand tumor cells produce factors that induce the formation of a neural network, a process called neoneurogenesis [14] and on the other hand the newly formed nerves release neurotransmitters that affect tumor growth and migration [15,16]. For example, many cancer patients exhibit signs of stress and depression, that have an effect on the immune system and tumor growth [17]. The (direct) interaction between peripheral nerve cells and tumor cells is usually called the *neuro-neoplastic synapse* (Figure 2A) [18].

In addition to the formation of nerves supporting the formation of new tissue in the form of tumors, recent evidence has shown that cancer cells are able to actively migrate along nerves, this process is known as *neural tracking* and involves the cancer cells migrating along axons [19]. The most numerous types of nerve cells are neurons and Schwann cells. The nerve tissue consists of three different layers each consisting of different types of cells [20]. *Intra-neural invasion* is the name given to the invasion of the innermost layer of nervous cells, the endoneurium, and is an indicator of poorer outcomes for the patient than invasion of either the middle or outer layers, the endoneurium and perineurium [21]. This endoneurium layer consists of axons, Schwann cells, mast cells, resident macrophages, fibroblasts, and blood vessels. Schwann cells have been shown to be associated with cancer progression in many different types of cancers, such as pancreatic cancer, colon cancer, thyroid cancer and skin squamous cell carcinoma. The Schwann cells are also present at the terminal ends of nerve fibers, where they were found to be in contact with pre-cancerous cells that eventually progresses into invasive ductal adenocarcinoma [22,23,24].

The invasion of cancer cells along nerves or nerve tissue supporting the growth of cancers around nerves, is known as *perineural invasion* (PNI) [19]. This process is most common in cancers affecting organs that are extensively innervated, such as pancreatic cancer, head and neck cancer, prostate cancer and colorectal cancer [25,26]. PNI results in patients experiencing symptoms such as irritated skin, numbness, or paralysis and is an indicator of poor patient survival [27]. Neural bodies that are in close proximity to the tumors (such as post-ganglion neural series) can rapidly be influenced by, respond to, or influence the tumor microenvironment. The tumor micro-environment is also influenced by the sensory nerves which normally provide information to the central nervous system (CNS) [2]. Therefore, nerve tissue supports the growth and progression of cancer as well as providing a means for cancer to metastasize [5,15,28,29]. However, it is not yet known if the progression of cancer is related to the presence and action of existing neurons located in the region of the tumor or if the progression of metastasis requires the development of new neurons [13].

### 1.3. The Role of Nerve Growth Factors and Axon Guidance Molecules in Tumor-Neuron Interactions

In general, high levels of innervation in tumors correlate with a poor disease outcome. Indeed, tumor cells have the ability to produce substances that stimulate the growth and survival of nerve cells, such as the Nerve Growth Factor, or NGF, which belongs to a class of growth factors called *neurotrophins* [30,31]. These growth factors, together with glial cell-line derived neurotrophic factor family ligands (GFLs) and neuropoietic cytokines, form a family of proteins called *neurotrophic factors*, which control the growth and survival of newly formed neurons and the maintenance of mature ones. These growth factors can promote tumor growth [30] and inhibit aggregation of cancer cells, thus promoting tumor invasion, in a way that is not well understood yet [32]. Interestingly, it has been shown that NGF could also induce the migration of endothelial cells, thus enhancing angiogenesis (Figure 2B) [33]. In addition to these substances tumors also release axon guidance molecules. The term axon guidance denotes the process by which neurons send out axons along a very precise path in order to reach the correct targets. In fact, the tip of an axon (or *growth cone*) is equipped with receptors that can sense (gradients of) chemicals, called *guidance cues*, which “tell” them where to expand (Figure 2B) [34]. Recently, it was shown that many *axon guidance* molecules can also control neuronal survival and migration, and that they are present in many organs other than the nervous system [35]. Moreover, several studies suggest that axon guidance molecules may be involved in cancer, since they regulate cell migration and apoptosis [36]. In particular, three families of these proteins have been studied: the semaphorins, the slits and the netrins. Tumors then induce innervation around themselves [12,18] in a manner which is common to nerve regeneration in peripheral tissues [37].

### 1.4. ANS Effects on Tumor Cells

Although it has been empirically known for a long time that stress and depression affect the course of cancer, it is only recently that a more solid bio-chemical explanation of this hypothesis was found, after the discovery of the first neurotransmitter in the 20th century by Otto Loevi [38]. Initially, the effect that the nervous system has on cancer development was considered to be only “indirect”, through perineural invasion and modulation of immune function [39]. In fact, neurotransmitters regulate the cytotoxicity of T lymphocytes and natural killer cells [40] and induce leukocyte migration [15,41]. The consequent immunosuppression can favor tumor growth and progression, since it impairs the anti-tumor response [42,43]. It is this migratory effect of neurotransmitters on tumor cells that suggested a direct link between the nerves and tumor progression. In fact, one theory for the spread of a primary tumor to a certain organ proposes that circulating cancer cells are attracted by some factors (chemokines), which are largely released by some organs [44]. Hence, many studies have been produced showing that neurotransmitters such as noradrenaline, dopamine or substance P influence the migratory activity of cancer cells, perhaps through inducing a genetic change towards a more motile phenotype [40,45], or by simple chemotaxis (i.e., motion along a gradient of a chemical such as noradrenaline, [46]). Moreover, some neurotransmitters such as histamine, serotonin and angiotensin also induce tumor growth [15]. In summary, the effects that the nervous system has on tumors are either indirect (such as perineural invasion and immunosuppression) or direct (since neurotransmitters induce chemotaxis, migration and growth of cancer cells).

### 1.5. Propinquity with Angiogenesis and Lymphangiogenesis

The most evident similarity between the nervous system and the blood and lymphatic vasculature is that they follow similar patterns and are often found together throughout the body (especially in the tumor microenvironment). One explanation of this phenomenon has been provided by studies showing that guidance cues for axons also play a role in angiogenesis [47,48,49]. Both tissue types respond to the same patterning cues, forming similar branching patterns [47]. These patterning cues include axon guidance molecules. Actually, these systems are interconnected through several molecular signaling pathways that facilitate their development and activation in cancer [50,51].

A critical link between neural signals in the tumor microenvironment and angiogenesis was revealed in the work by Zahalka et al. studying mouse models of prostate cancer [52]. Their results showed that adrenergic signals promoted the angiogenic switch in prostate cancer while their inhibition using beta-blockers could either prevent or delay pro-angiogenic factors that promote tumor progression. More specifically, adrenergic nerves regulate the ability of new blood vessels to form a pattern capable of providing the tumor with enough nutrients through the new blood vessels. This occurs in the early stages of cancer [29]. Following up on these results, Zahalka et al. [52] established a correlation between adrenergic nerves and tumor metabolism. The authors showed that nerve-activated ADBR2 signaling in prostate endothelial cells resulted in reduced oxidative phosphorylation. This switch involves the expression of the gene *Adrb2*, the gene encoding the β_2_-adrenergic receptor. The authors suggest that the loss of this gene leads to an angiogenic switch that fuels cancer progression via the inhibition of oxidative phosphorylation [52,53].

Adrenergic nerves are also found in high numbers in the lymphatic system. These nerves regulate the flow of lymph [51]. The adrenergic signaling-dependent remodeling of the lymphatic system was found to occur in breast cancer models. Sloan and co-workers [54] demonstrated that chronic stress restructures lymphatic vessels to accelerate tumor cell dissemination. In their in vivo experiments using an orthotopic mouse model of breast cancer they revealed that chronic stress not only increased the density of the lymphatic vessels within tumors, but also increased the diameter of collecting lymphatic vessels that drain primary tumors toward distant lymph vessels. The aforementioned effect maintained even two weeks after stress was ceased, suggesting a stable, increased capacity for tumor cell dissemination via lymphatic routes under stressed conditions. The connection between the SNS and the lymphatic system increased the odds of a direct implication of SNS on lymph flow besides on its effect on lymphatic restructure. This remodeling was once again dependent on ADRB2 [54]. On the other hand, denervation has a direct effect on the lymphatic system by decreasing lymphangiogenesis and therefore inhibiting cancer progression [55].

In summary, angio-, lymphangio- and neoneuro-genesis are alike, in the sense that they have common regulation factors and all together promote metastasis. For instance, tumor cells secrete angiogenic factors (mainly VEGF) which initiate tumor vascularization, but at the same time tumor-innervating nerve cells release neurotransmitters which are proliferative or promigratory signals for the tumor cells. Furthermore, nerve fibers can constitute a kind of “route” for tumor cell to disseminate, perineural invasion, such as blood and lymphatic vessels [15]. Considering all these factors, the hypothesis has been advanced that tumors stimulate their own innervation in a process similar to angiogenesis and lymphangiogenesis. Moreover, neurogenic and angiogenic factors have overlapping functions. For example, in [12] it was reported that NGF has angiogenic effects, and that the axonal attractant netrin-1 is also an angiogenic factor. In turn, VEGF seems to be a chemoattractant for neural progenitors. It is then reasonable to think that more factors are involved in neoneurogenesis, as it happens for angiogenesis.

### 1.6. Neurotransmitters and Tumor Cells

Neurotransmitters are usually short-lived chemicals which transport signals through a synapse from one neuron to another “target”. They are packed into vesicles and stored in the axon terminal, on the presynaptic side of a synapse. Neurotransmitters are then released in response to a threshold action potential and diffuse across the synaptic cleft, where they bind to specific receptors in the membrane on the postsynaptic side of the synapse (Figure 2C). Many neurotransmitters are synthesized from simple precursors such as amino acids, which are generally readily available and require only a few steps to be converted into neurotransmitters. Tumor cells express many neurotransmitter receptors such as G protein-coupled receptors, or GPCRs, also known as *serpentine receptors* [56]. Neurotransmitters act as ligands for these kinds of receptors, and can induce several behavioral changes in tumor cells, mostly increasing their proliferation and/or migration. Such effects are summarized in [57]. It seems that tumors are also able to produce neurotransmitters, maybe (only) after neuroendocrine differentiation. In fact, it has been observed that prostate cancer cells can acquire neuroendocrine characteristics, perhaps as an adaptive reaction against therapeutic agents [58,59,60].

### 1.7. Cancer Development and Stress

The release of neurotransmitters has been found to be altered by stress or depression [61]. This was observed in studies where it was noted that the induction of catecholamine by cellular stress led to increased levels of neurotransmitters such as noradrenaline [62]. This cellular stress stimulus is associated with increased incidence of cancer in a variety of solid tumors, including ovarian, prostate, breast, and pancreatic cancer [55,63,64,65,66]. For example, the development of pancreatic cancer in stressed mice is decreased by the removal of the bilateral adrenal gland. Since noradrenaline signals through α-adrenergic and β-adrenergic receptors and stress results in nerves releasing noradrenaline, β-adrenergic agonists have been found to stimulate the stress induced initiation and progression of cancer [66]. Inhibiting the signaling of noradrenaline by non-selective β-blockers inhibited lymph node metastasis [54,55]. Deletion of genes encoding β2- and β3-adrenergic receptors (ADRB2, ADRB3) in adult endothelial cells inhibited cancer progression [63]. Moreover, several pathways function downstream of signaling β-adrenergic receptors. These include cAMP response element-binding protein (CREB), muscarinic Ach receptor-1(M1R) activation of the phosphoinositide 3-kinase-mitogen-activated protein kinase (PI3K–MAPK) pathway and muscarinic acetylcholine receptor subtype 3 (M3R) activation of the WNT signaling pathway [67]. Mouse models of glandular hyperplasia have been used to demonstrate how increased nerve signaling can promote cancer progression by stimulating the superior cervical ganglion of the mouse, which stimulates the salivary glands [68,69].

### 1.8. Nerve Growth Factor and Alternative Splicing

As it has already been mentioned in Section 1.3, Nerve Growth Factor (NGF) is a neurotrophic growth factor that binds to two different receptors. One is a high affinity receptor, Tropomyosin-related kinase A (TRKA) while the other is the low affinity receptor p75 neurotrophin receptor (p75NTR). NGF has been shown to play a protective role, promoting the survival of nerve cells. It also acts as a regulator of neurotransmitters and neuropeptides synthesis [70]. Decreased NGF activity negatively affects sensory perception [71], while its overexpression is related to increased neuronal plasticity in the adult nervous system [72]. NGF also acts on cells in the endocrine and immune system, implicating it in neuro-immuno-endocrine interactions [73]. NGF also provides the first case of alternative splicing to be discussed in this review. NGF is synthesized as a precursor pro-protein (proNGF), which is twice the size of the mature protein. NGF is a relatively small protein of 118 amino acids [72,74]. In mice, alternative splicing gives rise to two splice variants (proNGF-A and proNGF-B). These isoforms differ in that the A variant has a 66 amino acid N-terminal portion [75]. These variants arise from the use of alternate promoters [76]. While both variants promoted neurotrophic responses when both receptors were active, the inhibition of either TrkA of p75NTR demonstrated that these two variants were uniquely related to the activity of the two receptors. ProNGF-A promoted survival of neurons when TrkA was blocked, while ProNGF-B had the effect of causing the cells to differentiate when the p75NTR receptor was blocked. Therefore, the pro or anticancer signaling of these variants may be the result of the ratio of the different receptors on the cell surface, in combination with the different ratio of variants present [77]. Pro-NGF has the added ability to bind to a p75NTR-Sortilin receptor complex, allowing it to initiate pro-apoptotic signaling [78,79].

### 1.9. Other Neurotrophins

Another important neurotrophin is the glial cell line-derived neurotrophic factor (GDNF) which binds to the GDNF family receptor-α2 (GFRα2) receptor [80,81]. These neurotrophins and their receptors are responsible for tissue remodeling during development in a process known as *lobulation*. The recruitment of nerves is an absolute requirement for this process [80,81]. For example, during gland development a branching network of ducts is required to increase the surface area of the metabolically active tissue to allow for the secretion of enough hormone. During this process the end bulbs of the ducts release neurturin. Neurturin is another member of the GDNF family of neurotrophins. The release of neurturin leads to axonal growth and the expression and secretion of GFRα2 [80] and acetylcholine, which induces epithelial progenitor cells to grow and multiply to form branches and acini [80,82,83,84]. Further contributions of neurotrophins to cancer were observed in transgenic mouse models of pancreatic cancer. The preneoplastic stage of pancreatic cancer in transgenic mice is accompanied by an increase in the expression of various neurotransmitters and their corresponding receptors. The levels of neurotrophin in these mice continued to rise as the disease progressed [85].

## 2. The Role of Nerve Tissue in Different Cancers

As previously mentioned, it is not currently known whether the existing nerve fibers surrounded by the tumor are sufficient for the development and progression of cancer or it requires newly formed neurons. This is because the role played by nerve tissue in cancer progression and metastasis is not fully understood. Although pre-existing peritumoral neurons are likely to be sufficient for tumor spread, recruitment of neurons in the close proximity of a tumor may increase the propensity of tumors to metastasize. Increased neuronal density and/or the presence of intra-tumoral neurons should therefore be regarded as an additional unexplored pathway rather than as a necessity for metastasis.

Significant progress has recently been made in understanding the pathophysiology of the neuronal system in cancer biology [12,13,28,29], and this can be attributed principally to two main factors: the discovery of the role that neurotrophic growth factors play in the formation of a neural network (neoneurogenesis) and the identification of neuroproteins as new potential biomarkers that also act as an important regulator of tumor growth and dissemination [86]. Recent studies revealed that the activation of beta-adrenergic signaling, that is associated with increased chronic stress, regulates cancer progression in several cancer types (breast, pancreatic and lymphoblastic leukemia) [87,88,89]. Furthermore, Madeo et al. investigated the role of tumor-produced exosomes on tumor innervation and more specifically those exosomes containing the axonal guidance molecule ephrinB1 [90]. These exosomes that contain epherinB1 have the ability to potentiate neurite outgrowth in vitro and tumor innervation in vivo [90]. In the following, specific experiments and findings are collected and summarized on the observed connections between nerves and specific cancer types.

### 2.1. Prostate Cancer

An ideal example highlighting the role that the nervous system could play in tumor development, progression and metastasis is prostate cancer. As it is already known the prostate gland is fed by an anatomically distinct set of sympathetic and parasympathetic neural inputs. Although it was well established that perineural invasion by prostate cancer cells correlated with poor prognosis [91,92] the role of nerves in prostate cancer development and progression was underappreciated until recently. The findings by Magnon et al. who used a series of human prostate xenograft and transgenic mouse models credited the role of neoneurogenic responses in initiating prostate cancer development through adrenergic fibers (rather than from the sympathetic nervous system) [16]. Additionally, through the release of acetylcholine that stimulates Chrm1 muscarinic receptors on stromal cells (fibroblasts and smooth muscle cells) they reveal the importance of the parasympathetic nervous system in the ability of prostate cancer cells to invade, migrate and metastasize [16,93]. The aforementioned work pinpoints tumor-induced neoneurogenic processes as a potential therapeutic target for prostate cancer. In a similar manner, Lolas and co-workers developed the first mathematical model confirming tumor induced neoneurogenesis as a target for cancer drug development [94]. In their mathematical model investigated stress as a regulator of cancer etiopathogenesis predicting that stress can directly affect primary tumor growth through the release of neurotransmitters.

### 2.2. Pancreatic Cancer

In humans the development of pancreatic cancer is accompanied by increased expression of neurotrophin in the stromal compartment and an increase in the expression of neurotrophin receptor in the epithelial compartment [95,96]. It is currently not known how the increased levels of these molecules interact in the different compartments, but this increase in expression is associated with patient’s poor survival and treatment outcomes [95,96]. The development of pancreatic cancer in stressed mice is decreased by the removal of the bilateral adrenal gland. Since noradrenaline signals through α-adrenergic and β-adrenergic receptors and stress results in nerves releasing noradrenaline, β-adrenergic agonists have been found to stimulate the stress induced initiation and progression of pancreatic cancer [66]. Meanwhile, beta-adrenergic signaling blockade could potentially slow or prevent pancreatic tumor growth and invasion and therefore complement existing treatments. Apart from stimulating cancer, increased nerve signaling, and activity can prevent cancer progression. This was observed in pancreatic cancer, where parasympathetic nerve activity leads to slower cancer progression. This was demonstrated by the truncation of parasympathetic nerve signaling by transection of the vagus nerve, leading to accelerated tumor growth [97,98]. The progression of pancreatic cancer can also be inhibited by cholinergic signaling in mice xenograft models [98].

### 2.3. Breast Cancer

In human xenograft patients and transgenic mouse models, increased activity of cholinergic nerves leads to decreased rates of cancer progression [99]. Breast tissue consists of epidermal tissue, derived from the apocrine glands, and therefore receives nerve impulses in the same way as skin while having a similar pattern of innervation [100,101,102]. Recurrence of breast cancer is associated with the density of adrenergic nerves in the original tumor specimens taken from the patient prior to treatment [99].

Meanwhile, Sloan and co-workers revealed that by silencing β_2_AR expression in breast cancer cell line (MDA-MB-231) blocked the metastatic effect that stress may have [103]. These results identified tumor cells as an additional target of stress-induced βAR signaling. More specifically they resulted in knockdown studies where that β_2_AR knockdown through isoproterenol treatment shifted MDA-MB-231^HM^ cells towards a mesenchymal morphology. Therefore, decreased activation of βAR induced pathways led to differentiation and decrease cancer cell behaviours. Meanwhile, in a similar study the authors showed that α_2_-adrenergic blockade enhances breast cancer progression in a similar manner as chronic stress does [89].

There are high levels of brain-derived neurotrophic factor (BDNF) present during the development of the mammary gland. BDNF activates the Tropomyosin receptor kinase B (TrkB) receptor on sensory neurons. This leads these nerves to form new nervous tissue in the ductal tree of the developing breast [104]. These findings indicate that the deletion of the genes encoding BDNF or TrkB, could potentially inhibit the formation of nerve tissue in the developing breast. [105].

### 2.4. Gastric Cancer

In mice gastric cancers show an increased expression of acetylcholine receptor 3 (M3R)3. The growth of gastric cancer in these mice can be slowed by the deletion of M3R in gastric epithelial cells [67]. The development of gastric adenocarcinoma in wild type mice is accompanied by the overexpression of NGF in the epithelial cells of the gastric tissue. The higher levels of NGF resulted in increased innervation of the gastric mucosa [67]. Another important pathway that is involved in the nervous system influencing the development of gastric cancer is the WNT signaling pathway. The Wnt signaling pathway is involved in embryonic development and is known to be associated with cancer where it plays a role in rapid cell division and migration by preventing β-catenin degradation [106] Increased WNT signaling was also detected in gastric cancer [11]. In this case increased WNT signaling corresponds with increased nerve density [11]. It is important to note that higher levels of WNT signaling was found to correlate with gastric cancer specimens at more advanced stages of disease [11]. WNT signaling is required for the regeneration of tissue in the tip of digits due to its ability to regulate neurotrophic factors. It is suspected that WNT signaling may play a similar role in the development of cancer [11,107].

### 2.5. Malignant Gliomas

The central nervous system (CNS) is known to modulate and promote the development of glial cells through stimulating the formation of glial precursor cell proliferation [108]. Under certain conditions this can lead to the development and progression of malignant gliomas. This was established using experimental animal models [109,110,111]. The CNS is able to induce these effects through paracrine and electrochemical signaling. Growth factors are secreted from neurons and from glial cells, which can stimulate gliomas [109]. Malignant gliomas can also form electrically active components of the neuronal network through synapses connecting neurons and cancer cells [110,111,112]. Malignant glioma cells are connected to other malignant glioma cells through gap junctions. This allows neuronal signaling to be extensively conducted through nerve tissue and malignant gliomas [110,111].

In brain cancers there is a loss of adrenergic nerves and Schwann cells [113]. Like hematologic cancers, and unlike cancers arising in epithelial tissues, an increase in the activity of adrenergic nerves is accompanied by a decrease in the development and progression of cancer, rather than promoting it. Tumor growth in brain cancers can also form a network of excitatory synapses that can drive tumor progression [112]. However, the work by Zeng et al. showed that this phenomenon is not limited to brain tumors. More specifically, the authors showed that breast cancer cells that spread to the brain can act such as neurons forming similar excitatory synapses [114]. In both cases this new neural network contains glutamatergic nerves, these tumors can produce metabotropic glutamate which acts on glutamate receptors known as N-methyl-d-aspartate receptors (NMDARs) [114]. Outside of the CNS, tumor related expression of NMDARs is associated with increased aggressiveness and malignancy [115]. All the above cases highlight the adaptive capabilities that cancer cells have in different environments.

### 2.6. Nerves in Head and Neck Cancer

As in all the other solid tumors, the microenvironment of head and neck tumors includes nerve fibers that arise from the peripheral nervous system while the density of these nerves and perineural invasion has been associated with poor clinical outcomes. From the molecular point of view, it is well known, that the TP53 tumor suppressor gene remains the most-commonly mutated gene in head and neck cancer. Driven by this observation, in a recent study Amit and co-workers investigated the mechanisms of cancer-nerve interactions based on the p53 suppressor gene function [116]. Their findings highlighted the crosstalk between the peripheral nervous system and head and neck tumors. More specifically, they revealed that cancer cells trigger a phenotypic shift in which sensory nerves differentiate into adrenergic neo-neurons. They showed that in p53-deficient tumors, a miRNA-based mechanism drives neuronal responses to environmental cues and decides the fate of cancer-associated neurons. In other words, cancer cells can orchestrate an axonal sprouting as well as autonomic reprogramming of existing nerves as a consequence of miRNA shuttling.

Another model studying the dynamic interaction between nerve and head and neck cancer was proposed by Scanlon et al. [117]. In their data, they showed a novel mechanism where the neuropeptide galanin (GAL) from nerves stimulates its G protein-coupled receptor, GALR2 on cancer cells to induce nuclear factor of activated T cells, cytoplasmic, calcinneurin-dependent-2 (NFATC2)-mediated transcription of pro-inflammatory mediators (such as cyclooxygenase-2 (COX-2)) and neuropeptides from tumor cells leading to invasion and tumor progression. Following a similar feedback manner, GAL secreted by cancer cells induces neurogenesis and PNI.

### 2.7. Nerves in Haematological Malignancies

Despite nerves arising from epithelial tissue, they do play a role in hematological cancers. Blood cancers arise due to improper functioning of hematopoietic stem and progenitor cells (HSPCs). The activity of these cells is regulated by their microenvironment. Adrenergic nerves are found at a high density in the bone marrow [118,119,120]. The density of adrenergic nerves decreases significantly with age. This leads to decreased levels and activity of HSPCs and the loss of HNSPCs can promote malignancy [119,120].

### 2.8. Reactivation of Nerve-Mediated Pathways

Recent studies have shown that cancers are able to reactivate nerve dependent pathways and that this reactivation is able to contribute towards the growth and progression of the tumor. These neural pathways were previously active during development. Following the completion of organ or glandular development there is a decrease in the levels of NGF accompanied by a decrease in the formation of new nerves [81]. In the transgenic mice model of pancreatic cancer previously discussed in Section 3.3, it was noted that there were elevated levels of neurotransmitters, their receptors and neurotrophin present. This leads to the reactivation of neural pathways and an increase in the development of new nerve tissue. As the expression of these various components continued to rise the disease continued to progress [85]. As the pancreatic cancer advanced to a pancreatic ductal adenocarcinoma stage there was a concurrent increase in nerve density, with a higher percentage of these new nerves being adrenergic and expressing higher levels of NGF. It is important to remember that these new neural pathways arose from reactivated neural tissue that was reactivated in the early stages of tumor development [121]. The reactivation of nerves is further implicated in the development of cancer through the WNT pathway. As discussed, this pathway initiates cancer development through increased signaling resulting in increased nerve density. This pathway is also involved in tissue regeneration. Regeneration of tissue involves pathways similar to those in organogenesis and embryogenesis. These include signals for epithelial proliferation and cell migration. Parasympathetic nerves regulate cell proliferation and the migration of calls to form the structures to give rise to organs. This is achieved through SOX2+ epithelial progenitor cells being activated through M1R signaling, resulting in cells with stem cell such as characteristics [84].

## 3. Alternative Splicing in Nerves and Cancer

### 3.1. Doublecortin and Doublecortin-Like Proteins

**Figure 3:**
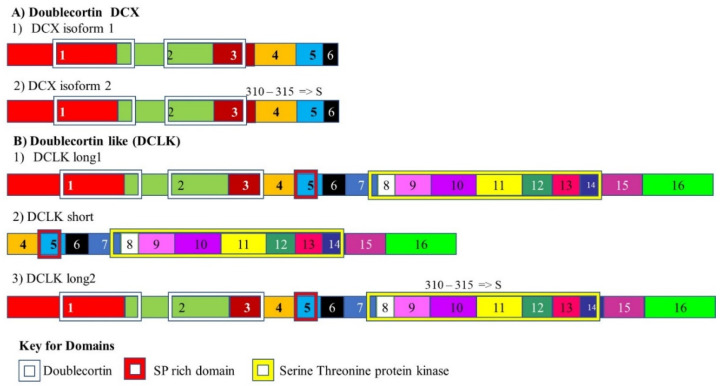
Splice variants of Doublecortin and Doublecortin-like. (**A**) Doublecortin (DCX) is alternately spliced to give rise to two isoforms. It interacts with microtubules in neuronal precursor cells and promotes cell division. It is expressed in the CNS and primary tumors. (**B**) Doublecortin-such as (DCLK) is alternately spliced to give rise to three isoforms. The short isoform of DCLK lacks both doublecortin domains and is generally only expressed in adults while the full-length form is expressed in developing embryos. However, the embryonic form is also expressed in tumors. The different numbered colored boxes represent exons. The size of the boxes represents the relative length of them. The frames represent functional domains within the different isoforms.

The protein doublecortin (DCX) is expressed in neuronal precursor cells and is associated with microtubules. It binds to the microtubules, stabilizing microtubule structures. This protein is expressed by these cells when they divide and by their daughter cells for some time after division. As such DCX is used as a marker for neurogenesis [122]. DCX positive cells are found in neurogenic regions of the CNS and in stroma of human primary prostate tumors. Doublecortin levels were also found to be increased in prostate cancer in mice [123]. These tumors also express other markers that are used to identify neural progenitor cells. These include: polysialylated-neural cell adhesion molecule (PSA-NCAM)18 and internexin [124,125]. The doublecortin domain binds to tubulin and requires a tandem repeat of the domain to function properly [126]. DCX is alternately sliced to give rise to two isoforms (Figure 3A). However, these isoforms are so similar in structure it is not thought that they play a significantly different role in cancer development and progression.

Doublecortin-like (DCLK) is so named because it also contains two DCX domains such as the original doublecortin protein. Like doublecortin, it also binds to microtubules. It is vital for correct embryonic neurogenesis. The DCLK gene is alternately spliced to give rise to at least three isoforms (Figure 3) [127]. The short isoform of DCLK lacks both doublecortin domains, implying that it cannot interact with the cytoskeleton. The shorter isoform is generally only expressed in adults while the full-length form is expressed in developing embryos. The adult form had reduced autophosphorylation activity. However, both forms display the same kinase activity for myelin. During tumor development and progression, it has been observed that the embryonic form is aberrantly expressed in adults where it possibly plays a role in migrating neurons [128].

### 3.2. Fibroblast Growth Factor Receptor-1 (FGFR-1)

**Figure 4:**
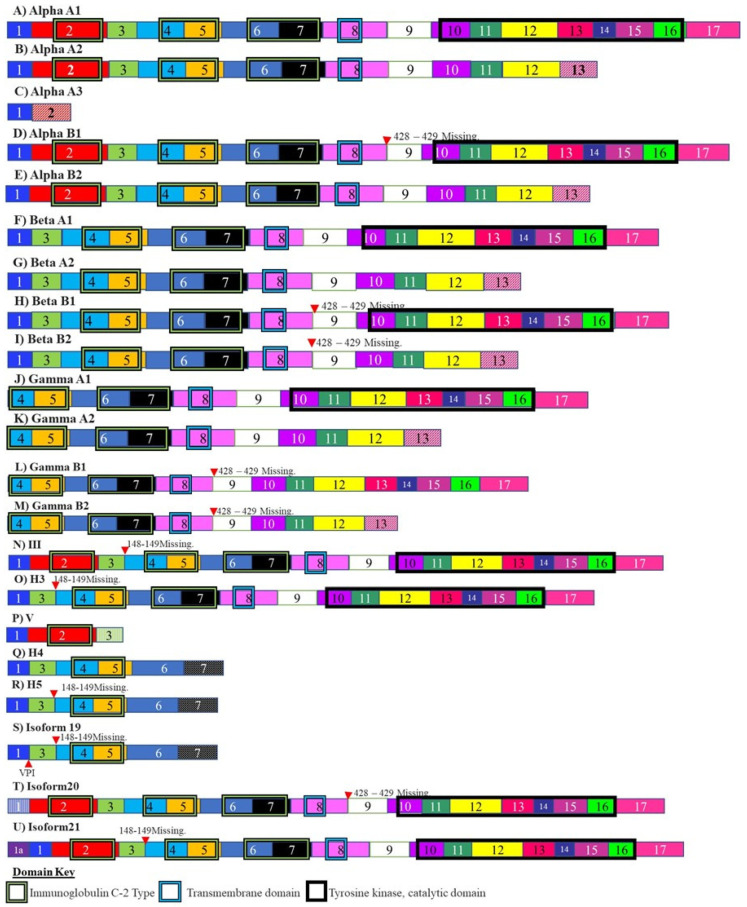
Isoforms of fibroblast growth factor receptor. These isoforms include the FGFR-1 isoform Alpha A3 which arises due to skipping of the alpha exon. This isoform has increased affinity for fibroblast growth factors and overexpression of this isoform results in increased malignancy. The different numbered colored boxes represent exons. The size of the boxes represents the relative length of them. The frames represent functional domains within the different isoforms.

Fibroblast growth factor receptor is a receptor tyrosine kinase that is an absolute requirement for correct embryonic development and patterning. FGFR-1 is known to be mutated or over-expressed in certain cancers [129]. Glioblastomas are made up of undifferentiated astrocyte precursors. These precursor cells are known to express up to 21 alternately spliced isoforms of FGFR-1 (Figure 4) [130]. These alternatively spliced variants of FGFR-1 include an isoform that arises due to increased skipping of exon 3, also known as the alpha exon (Figure 4). This results in an FGFR-1 isoform, that has a higher affinity for fibroblast growth factors [131,132]. When this isoform is over-expressed, there is an increase in the cases of malignancy as a result of increased growth stimulus. The splicing of the alpha exon is related to the multiple repressor elements that flank this exon [133].

### 3.3. Tyrosinase

**Figure 5:**
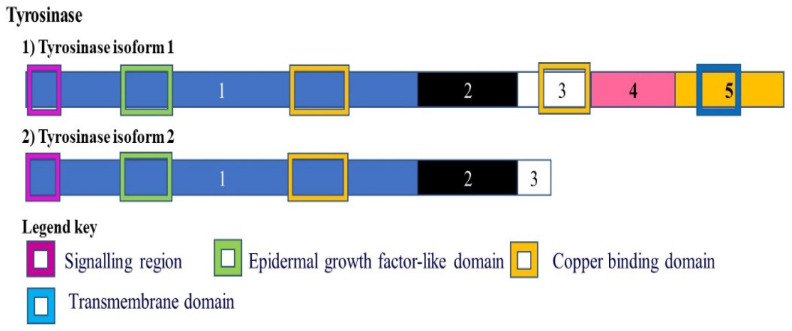
Alternative splicing of Tyrosine kinases in neurogenesis related to cancer: Tyrosinase is responsible for the synthesis of neuromelanin, dopamine and has been implicated in the spread of melanomas. Tyrosinase is spliced into two isoforms. One isoform lacks one of the coppers binding domain, resulting in decreased enzymatic abilities. The different numbered colored boxes represent exons. The size of the boxes represents the relative length of them. The frames represent functional domains within the different isoforms.

It has been suggested that the splicing of the tyrosine enzyme is under the control of neural splicing factors. This is because during normal embryonic development melanocytes are derived from the neural crest lineage. Malignant melanomas display reduced cellular pigmentation and differentiation. These melanomas also have multiple changes in alternative splicing including changes in the splicing of tyrosinase pre-mRNA [134,135]. Tyrosinase is also responsible for the synthesis of neuromelanin and catalyzes the formation of dopamine from tyrosine. Tyrosinase splicing gives rise to at least two isoforms, one of which lacks the second copper binding domain. This decreases its ability to catalyze reactions [136]. The expression of the resulting isoform could lead to loss of pigmentation (Figure 5) [134].

### 3.4. Tropomyosin Receptor Kinase B (TrkB)

**Figure 6:**
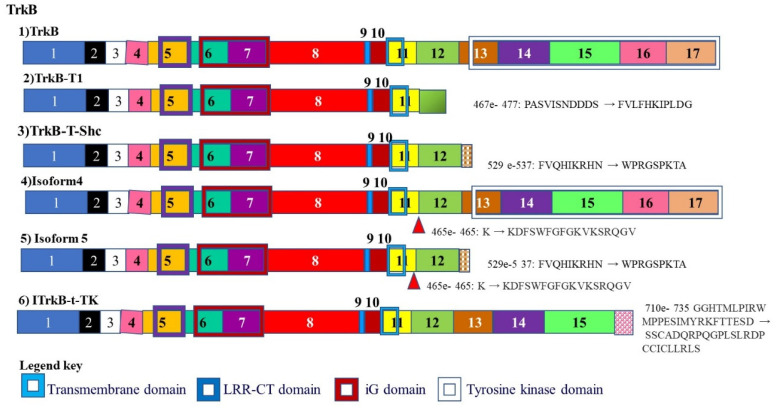
Alternative splicing of Tropomyosin receptor kinase B (TrkB): TrkB plays a role in synaptic survival and plasticity and is alternately spliced to give rise to an isoform that lacks the tyrosine kinase domain. It therefore lacks the ability to initiate intracellular signaling.

Trk receptors are a group of tyrosine kinases that play a role in synaptic survival, plasticity. One of the members of this group TrkB, is alternately spliced to give rise to two alternately spliced isoforms. An alternately spliced isoform of this receptor TRKB-T1 lacks the tyrosine kinase domain but possess all the extracellular domains and the transmembrane domain. This means it is able to interact with signals but not transmit intracellular signals and induce neuron survival and maintain plasticity (Figure 6) [137].

### 3.5. Amphiphysin II

**Figure 7:**
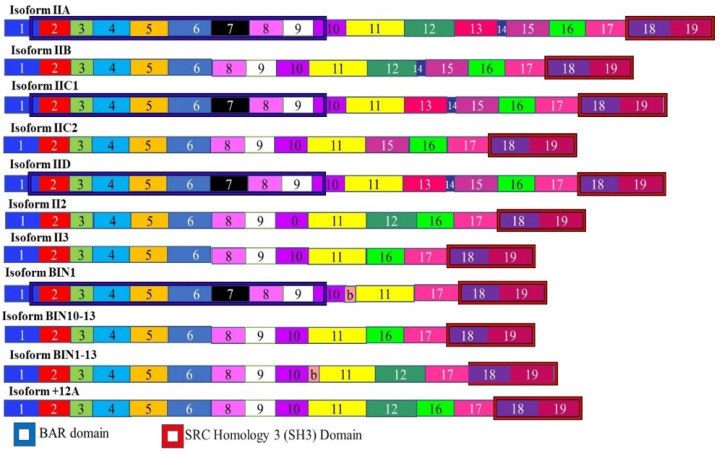
Isoforms of Amphiphysin II. The Amphiphysin II mRNA is alternately spliced to give rise to 11 isoforms. Different tissues express different ratios of these isoforms. The BIN1 isoform acts as a tumor suppressor and lacks exons 12A, 12B, 12C, and 12D. The inclusion of these exons results in an isoform that promotes cancer. The different numbered colored boxes represent exons. The size of the boxes represents the relative length of them. The frames represent functional domains within the different isoforms.

Amphiphysin II is alternately spliced to give rise to at least seven alternatively spliced isoforms (Figure 7). The alternate splicing and expression of these isoforms varies between different tissues. This includes muscle and brain specific splicing patterns. The splicing of Amphiphysin II changes in different cancers and was first noted in melanoma [138]. In the brain, the protein is expressed at high levels and is concentrated at the cell surface in nerve terminals. It is thought to play an important role in synaptic vesicle endocytosis [139,140]. The BIN1 isoform is believed to function as a tumor suppressor. BIN1 is produced by skipping exons 12A, 12B, 12C, and 12D. These exons are included in isoforms which are brain specific. It has been found that these brain specific isoforms have been found in certain melanomas and the BIN1 isoform is spliced to contain these exons in these melanomas, resulting in the loss of tumor suppressor activity [141]. It is thought that neuron-specific splicing factors may be responsible for these aberrant splicing events.

### 3.6. BHC80 and GIT1

**Figure 8:**
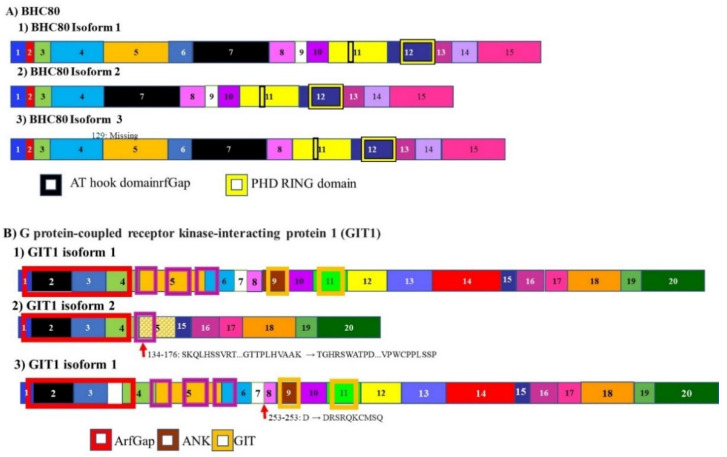
Alternative splicing in neuroendocrine prostate cancer progression: BHC80 and GIT1. (**A**) The histone demethylase BHC80 is alternately spliced to give rise to multiple isoforms. Only three are represented here. Two isoforms BHC80-1 and BHC80-2. Levels of BHC80-2 are expressed at higher levels in neuroendocrine prostate cancer (**B**) alternative splicing of G protein-coupled receptor kinase-interacting protein 1 (GIT1) gives rise to 3 isoforms. In the development of neuroendocrine carcinoma, the A isoform is upregulated, while the C isoform is downregulated. These isoforms control the splicing and expression of different populations of mRNA. The different numbered colored boxes represent exons. The size of the boxes represents the relative length of them. The frames represent functional domains within the different isoforms.

The histone demethylase BHC80 is alternately spliced to give rise to two isoforms, BHC80-1, and BHC80-2 (Figure 8A). BHC-80 is a member of the BHC complex. This complex inhibits the transcription of neuron-specific genes. It acts by modifying chromosomes by demethylating specific sites on histones. The expression of various BHC80 isoforms is associated with prostate adenocarcinoma progressing to neuroendocrine prostate cancer. It was determined that the progression from the milder adenocarcinoma to the more lethal neuroendocrine carcinoma, is accompanied by a shift in the levels of these transcripts with the levels of BHC80-2 being higher in neuroendocrine carcinoma prostate cancer [142]. This isoform localizes to the cytosol where it plays a role in the cell not associated with histones modification. Since it cannot modify chromosomes, it cannot suppress neuron specific genes. BHC80-2 activates the MyD88-p38-TTP pathway. This pathway stabilizes the mRNA coding for multiple tumor-promoting cytokines leading to increased cell proliferation and tumor progression [142].

Another gene whose mRNA splicing changes during the progression from adenocarcinoma prostate cancer to neuroendocrine carcinoma prostate cancer, is the G protein-coupled receptor kinase-interacting protein 1 (GIT1). The mRNA for this gene is alternately spliced to give rise to 3 isoforms (Figure 8B). The GIT1-A isoform is upregulated while the GIT1-C isoform is downregulated. These two isoforms regulate different populations of RNA Splicing of GIT1 mRNA driven by SRRM4. The neuroendocrine carcinoma prostate cancer promoting GIT1-A regulates genes associated with morphogenesis, neural function, environmental sensing via cell-adhesion processes, and epigenetic regulation. Consistent with previous transcriptomic analyses, opposing functions of GIT1-A and GIT1-C in the stability of focal adhesions was reported, whereby GIT1-A promotes focal adhesion stability. In summary, this previous study is the first to report that alternative RNA splicing of the GIT1 gene is associated with t-NEPC and reprograms its function involving FA-mediated signaling and cell processes, which may contribute to t-NEPC development [143].

### 3.7. TDP-43

**Figure 9:**
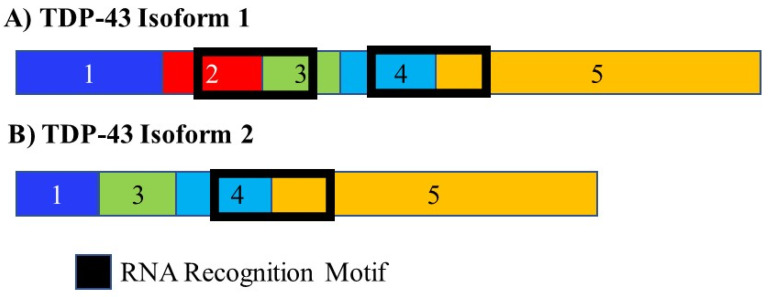
Alternative splicing of TDP-43. The RNA binding protein TDP-43 is alternately spliced to give rise to two isoforms. It controls the splicing of multiple mRNAs. Many of the RNA targets of TDP-43 play a role in neurodegenerative disease. It also controls its own splicing and gives rise to an isoform that either cannot be translated and is degraded or only contains a single RNA recognition domain, changing the population of RNA molecules it is able to interact with. The different numbered colored boxes represent exons. The size of the boxes represents the relative length of them. The frames represent functional domains within the different isoforms.

The RNA binding protein TAR DNA binding protein 43 kDa (TDP-43) was found to bind thousands of RNA molecules extracted from brain tissue. Mutations in TDP-43 can result in amyotrophic lateral sclerosis. Many of these target’s code for transcripts encoding proteins that play a role in neurodegenerative disease. In mice, depletion of TDP-43 results in splicing changes in 965 genes most of which contain very-long introns and are involved in synaptic activity. TDP-43 regulates its own expression through alternate splicing. It promotes the inclusion of a 3′ intron. This leads to an RNA molecule that cannot be translated and which is degraded, decreased TDP-43 expression and alterations in alternate splicing (Figure 9) [144].

### 3.8. The “Brain Cancer Paradigm”

Brain cancer diagnosis and classification is required for diagnosis and treatment of gliomas. In order to achieve this, both diagnostic and prognostic biomarkers need to be identified. These biomarkers would ideally be circulating and be able to not only diagnose cancer but also stratify it based on stage and progression. As indicated previously, the expression of alternatively spliced isoforms related to neuron expansion in developing cancers, changes as the disease progresses. This has been demonstrated in gliomas. The splicing profiles identified in this study were able to classify the glioma by type, sub-type, and tumor grade [145]. More generally, since the interactions between tumor and nerve cells are widely documented and accepted in brain cancer (particularly in glioma) [112,114], we can consider this well-known case as a “paradigm” of how cancer-neural interactions work and how treatment targeting this connection could be carried out.

### 3.9. Implications for Treatment

The change in alternative splicing patterns that occurs as tumors develop and progress, indicates that these isoforms are involved in processes that are important for the continued growth of the tumor. This implies that they will make excellent targets for the development of future drug treatments. While the therapeutic potential of denervation is well known, the presence of these alternatively spliced isoforms provides a means whereby this can be accomplished at the molecular level, with little or no surgical intervention. Crude forms of chemical denervation currently rely on the use of β-adrenergic antagonists or blockers, which have been shown to reduce the progression of cancer and improve patient survival [146].

**Figure 10:**
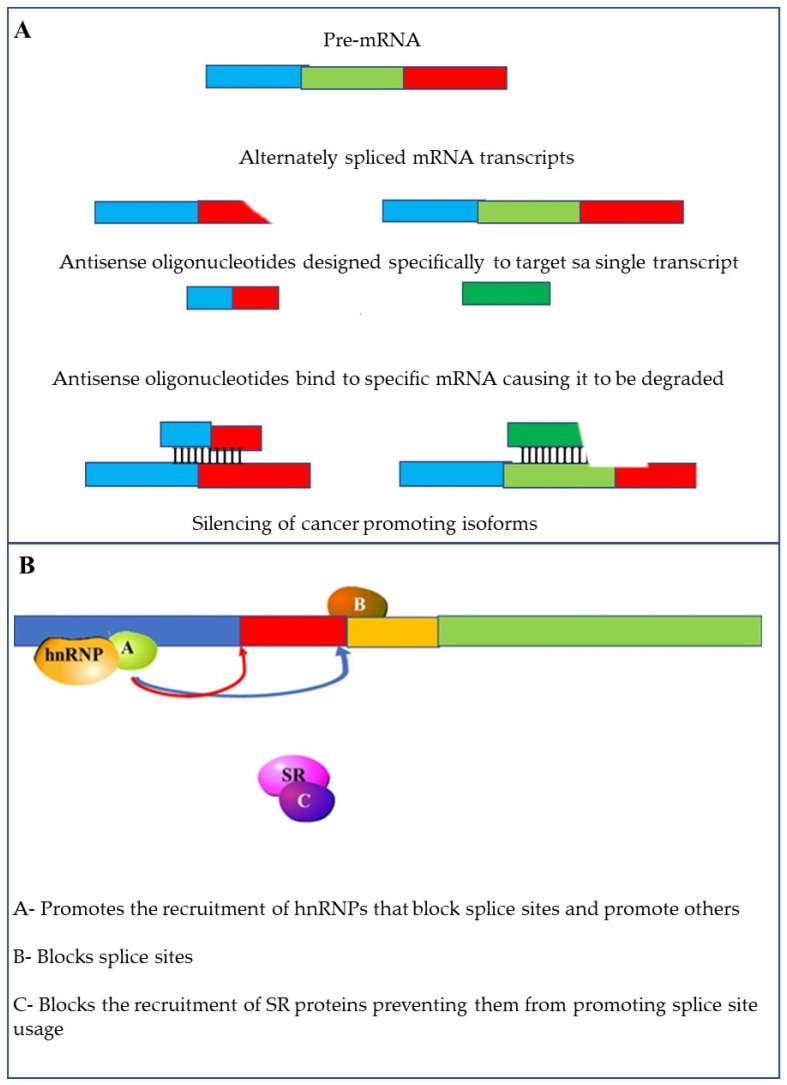
Methods to target alternative splicing: (**A**) altering splicing using specific antisense oligonucleotides (ASOs) using small oligonucleotides that are complementary to the target mRNA; (**B**) small molecular inhibitors can be used to interfere with the activity of splicing factors such as hnRNPs and SR proteins; Other small molecule inhibitors can block access to splice sites.

Methods that can be used to alter alternative splicing include the use of specific antisense oligonucleotides (ASOs). The RNA based ASO drug Spinraza™, is a therapy that affects splicing and has received FDA approval [146]. This demonstrates that ASOs can successfully be used to target splice variants for therapy. These short (15 to 30 nucleotides) modified RNAs can specifically target mRNAs and influence splicing events. This can be accomplished through splicing switches by targeting and blocking splice sites, enhancing sequences or silencing sequences [147] (Figure 10A).

Another way in which splicing can be influenced is through the use of small molecules that affect splice site selection and splicing efficiency. These molecules can also be used to target the spliceosome by preventing the recruitment of U4/U5/U6 tri-snRNPs [148,149]. Finally, these molecules can be used to target splicing factors. This can be achieved by inhibiting the phosphorylation of SR-proteins [148] or reducing the expression and increasing the ubiquitination and degradation of other splicing factors (Figure 10B) [150].

## 4. Conclusions

The long-held underestimation of the role neurons play in cancer initiation, growth and dissemination is changing rapidly. The newly discovered effects of neural-signaling, nerve growth factors and axon guidance molecules in regulating cancer initiation and progression open new unexplored ways in the emerging area of cancer neurobiology. It is known that developing tumor cells require new nerve tissue in the same way as they require the development of new blood and lymphatic vessels. This may be the principal cause of neoneurogenesis around the tumor, which in turn elicits specific reactions from different tumor types. Of particular interest is the fact that the development of new nerve tissue requires changes to the population of splice variants in the developing tumor and new nerve tissue. From a broader perspective, the identification of splicing variants associated with the growth of new nerve fibers, required for cancer progression, provides new targets for drug development as well as diagnostic biomarkers. Additionally, these isoforms can be used as tools for further research into the role played by the nervous system into the development and progression of cancer. Thus, cancer neurobiology appears as a new field for more basic and clinical research that could give rise to new innovative strategies for the diagnosis, prognosis, and treatment of cancer.
